# Clinical profile and evolution of patients with juvenile-onset Behçet’s syndrome over a 25-year period: insights from the AIDA network

**DOI:** 10.1007/s11739-021-02725-9

**Published:** 2021-04-09

**Authors:** Jurgen Sota, Donato Rigante, Giuseppe Lopalco, Giacomo Emmi, Stefano Gentileschi, Carla Gaggiano, Luisa Ciarcia, Virginia Berlengiero, Mariam Mourabi, Nicola Ricco, Sara Barneschi, Irene Mattioli, Gian Marco Tosi, Bruno Frediani, Maria Tarsia, Gerardo di Scala, Antonio Vitale, Florenzo Iannone, Claudia Fabiani, Luca Cantarini

**Affiliations:** 1 Research Center of Systemic Auto Inflammatory Diseases, Behçet’s Disease and Rheumatology-Ophthalmology Collaborative Uveitis Center, Rheumatology Unit, Policlinico “Le Scotte”, Department of Medical Sciences, Surgery and Neurosciences, University of Siena, Viale Bracci 16, 53100 Siena, Italy; 2 Department of Life Sciences and Public Health, Fondazione Policlinico A. Gemelli IRCCS, Rome, Italy; 3 Università Cattolica Sacro Cuore, Rome, Italy; 4 Rheumatology Unit, Department of Emergency and Organ Transplantation (DETO), University of Bari, Bari, Italy; 5 Department of Experimental and Clinical Medicine, University of Florence, Florence, Italy; 6 Clinical Pediatrics, Department of Molecular Medicine and Development, University of Siena, Siena, Italy; 7 Ophthalmology Unit, Department of Medicine, Surgery and Neuroscience, University of Siena, Siena, Italy

**Keywords:** Behçet’s syndrome, Uveitis, Childhood, Pediatric age, Personalized medicine

## Abstract

Behçet’s syndrome (BS) represents an understudied topic in pediatrics: the main aims of our study were to characterize demographic and clinical features of a cohort of BS patients with juvenile-onset managed in three tertiary referral centers in Italy, evaluate their evolution in the long-term, and detect any potential differences with BS patients having an adult-onset. Medical records of 64 juvenile-onset and 332 adult-onset BS followed-up over a 2-year period were retrospectively analyzed and compared. Mean age ± SD of first symptom-appearance was 10.92 ± 4.34 years with a female-to-male ratio of 1.06:1. Mucocutaneous signs were the most frequent initial manifestations, followed by uveitis. Throughout the disease course, genital aphthae (76.56%) and pseudofolliculitis (40.63%) prevailed among the mucocutaneous signs, while major organ involvement was represented by gastrointestinal and ocular involvement (43.75 and 34.38%, respectively). No significant differences emerged for both mucocutaneous signs and specific major organ involvement between juvenile-onset and adult BS patients. After excluding nonspecific abdominal pain, juvenile-onset BS patients were less frequently characterized by the development of major organ involvement (*p* = 0.027). Logistic regression detected the juvenile-onset as a variable associated with reduced risk of long-term major organ involvement (OR 0.495 [0.263–0.932], *p* = 0.029). In our cohort, juvenile-onset BS resembled the clinical spectrum of adult-onset patients. Pediatric patients with a full-blown disease at onset showed a more frequent mucocutaneous involvement. In addition, patients with juvenile-onset seemed to develop less frequently major organ involvement and had an overall less severe disease course.

## Introduction

Behçet’s syndrome (BS) is a systemic multifactorial autoinflammatory disorder originally described with the triple symptom complex consisting of aphthous stomatitis, genital ulcers and uveitis [1–4]. Nevertheless, given its systemic nature, any organ can be potentially affected, with vascular, gastrointestinal and central nervous system involvement being the most commonly reported [5]. A complex pathogenesis [6] and a noticeable geographic variability under the epidemiological and clinical profile complicates the attempts to clearly define this complex syndrome. More in detail, BS is particularly prevalent in the Eurasian populations, along the ancient trading “Silk Route”, while it is rarely encountered in Western populations [7]. With regard to specific clinical manifestations such as gastrointestinal involvement and pathergy reaction, different frequencies in different countries have been reported [8–10]. It is also believed to have a different prognosis among certain subgroup of patients. In fact, male patients show a higher mortality, which is mainly due to central nervous system and large vessel involvement. Young males are also more prone to intraocular inflammation, which accounts for the highest morbidity [11, 12]. To further complicate the scenario of BS, even more unanswered questions raise in a pediatric setting. BS usually outbreaks during the third decade [13]. However, a small percentage of patients may experience their first symptom before the age of 16 years [14–19]. Due to the relapsing–remitting nature of this syndrome, many years can pass between the first clinical manifestation and the full-blown disease. Several studies have delineated different disease expressions and severity between children and adults [20–30]. With the aim of shedding light on some blind spots that characterize pediatric BS we herein report our multicenter experience on a large sample of juvenile-onset BS, focusing on their clinical spectrum and assessing differences with adult patients as well as potential predictors of disease severity.

## Methods

### Study design and participants

Medical records of 396 patients admitted between January 2015 and January 2017 in three tertiary referral rheumatologic centers working in Central-North and Southern Italy were retrospectively analyzed. The following demographic and clinical data were collected: gender, age, age at onset, disease duration, human leukocyte antigen (HLA)-B51, clinical manifestations at onset, and clinical manifestations throughout the disease course. Past as well as current therapeutic data were also included. Diagnosis of BS was established in accordance with the international study group criteria (ISG) [31] or according to the international criteria for Behçet’s disease (ICBD) [32]. Patients experiencing their first manifestation prior to the age of 16 years were considered to have juvenile-onset BS [33], whereas the term “pediatric BS” was reserved only to those patients receiving a diagnosis during childhood. The initial BS symptom was defined as the first clinical manifestation of BS. Pediatric Behçet’s disease criteria (PEDBD) [34] were retroactively applied to the cohort with juvenile-onset BS in order to define the subpopulation with full-blown disease at onset before the age of 16. Potential mimickers presenting with BS-like symptoms were ruled out. All patients were referred and regularly followed-up in the three referral centers in line with the best standards of care. Investigation for a possible major organ involvement was tailored according to the patient’s need and not by a pre-established protocol. In case of a strong suspicion of ocular, neurological or gastrointestinal involvement, patients were examined by a dedicated ophthalmologist, neurologist or gastroenterologist, respectively. Patients receiving biologic therapy also underwent chest X-ray film, Mantoux and/or QuantiFERON test, urine culture, markers for hepatitis B and C, serology for HIV, syphilis and *Toxoplasma gondii* to exclude any active or latent infection. Primary aim of the study was to characterize BS patients with juvenile-onset in terms of demographic and clinical features. Any potential differences from adult population were also examined. Further area of our research consisted in trying to detect predictors of disease severity as well as subanalyzing gender differences among juvenile-onset patients.

### Protocol approval

The study protocol was conformed to the tenets of the Declaration of Helsinki and was approved by the local Ethics Committee of the University of Siena (Reference No. 14951). Informed consent was obtained from patients or their legal guardians.

### Statistical analysis

Data were analyzed using IBMSPSS Statistics for Windows, version 24 (IBM Corp., Armonk, NY, USA). For univariate analysis, continuous variables were summarized with mean ± standard deviation (SD) or median ± interquartile range (IQR) as required, whereas categorical variables were reported as absolute frequencies (percentages). Categorical variables were analyzed by Pearson’s chi-square test or Fisher’s exact test as needed and post hoc test with adjusted residuals in case of contingency tables with dimensions greater than 2 × 2, while means were compared with Mann–Whitney *U* test. Normality distribution of our data was assessed with the Shapiro–Wilk test. Logistic regression model with stepwise backward selection was used to investigate potential predictors of development of major organ involvement, reporting odd ratios (ORs) and 95% confidence intervals (CIs). Major organ involvement was defined as ocular, neurologic, gastrointestinal or vascular involvement. Isolated headache was not considered a specific neurological feature of BS, unless other neurological features and/or positive magnetic resonance imaging findings were identified. Similarly, only endoscopically documented lesions were classified as related to BS gastrointestinal involvement and analyzed statistically. A *p* value lower than 0.05 was considered as statistically significant and all tests were two-sided.

## Results

### Demographic, clinical and therapeutic data of juvenile-onset BS

**Fig. 1:**
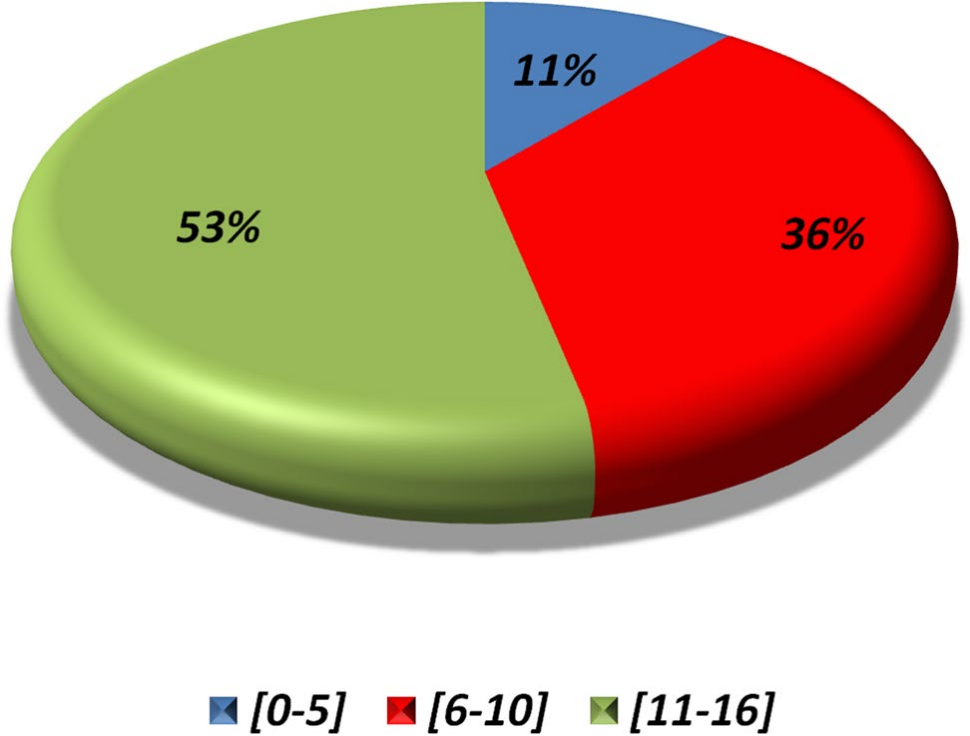
Distribution of age at onset for 64 patients with juvenile-onset Behçet’s syndrome (in accordance with the different age groups, expressed in years)

**Fig. 2:**
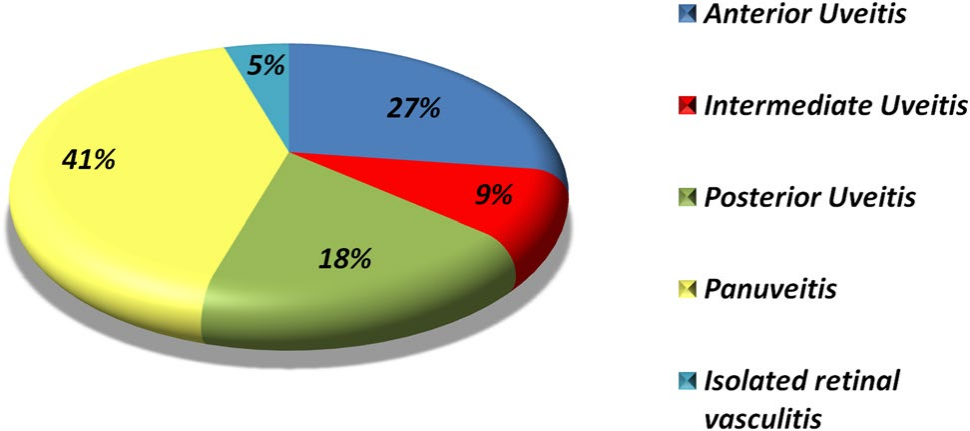
Anatomical patterns of intraocular inflammation classified according to SUN criteria for 64 patients with juvenile-onset Behçet’s syndrome evaluated in our study

Medical records of 64 juvenile-onset and 332 adult-onset BS patients fulfilling ISG or ICBD criteria at the moment of enrollment were retrospectively analyzed. The juvenile-onset population consisted of 33 male and 31 female patients (male-to-female ratio: 1.06:1). Mean ± SD age at the first symptom-onset was 10.92 ± 4.34 (median ± IQR 12.00 ± 8.50). The first symptom emerged after the age of 11 years in 34 patients. Figure 1 illustrates the distribution of age at disease onset. No cases of neonatal-BD were recorded. Eleven out of 64 patients (17.2%) with juvenile-onset had a family member affected by BS and one of them also had a second degree positive family history for Crohn’s disease. Unsurprisingly, the most frequent clinical manifestations at disease onset were oral aphthae (62/64, 96.87%) followed by genital aphthae (15/64, 23.43%), pseudofolliculitis or papulopustular lesions (12/64, 18.75%), and uveitis (9/64, 14.06%). Table 1 presents the demographic characteristics alongside with the clinical features at disease onset for juvenile-onset BS patients as well as their clinical manifestations occurring throughout the disease course. Overall, during BS course, genital aphthae (49/64, 76.56%) and pseudofolliculitis (26/64, 40.63%) prevailed among the mucocutaneous signs, while gastrointestinal signs (28/64, 43.75%) and intraocular inflammation (22/64, 34.38%) were the most common major organ-related manifestations. Gastrointestinal symptoms were usually mild, manifesting as abdominal pain and/or diarrhea without bloody stools. Uveitis was bilateral in 15 patients (68.18%) and unilateral in 7 (31.82), with panuveitis being the most commonly observed anatomical pattern (40.90%). Isolated headache was recorded in 22 patients. The anatomical pattern of uveitis, classified according to the Standardization of Uveitis Nomenclature criteria [35], is displayed in Fig. 2.

**Table 1:** Demographic characteristics and clinical manifestations for juvenile-onset patients at the onset of Behçet’s syndrome and throughout the disease course

Demographic features	Mean ± SD (median ± IQR) expressed in years
Male/female	33/31
Age (median ± IQR)	34.54 ± 23.61
Age at onset of the first symptom (median ± IQR)	12.00 ± 8.50
Disease duration (mean ± SD)	25.50 ± 14.80
HLA-B51, *N* (%)	30/60 (50.0%)
Clinical features at the onset	*N* (%)
Oral aphthosis	62 (96.88)
Genital aphthosis	15 (23.44)
Erythema nodosum	4 (6.25)
Pseudofollicular lesions	12 (18.75)
Papulopustular lesions	2 (3.13)
Arthritis or arthralgia	8 (12.5)
Uveitis	9 (14.06)
CNS signs	0 (0.0)
Gastrointentinal involvement	2 (3.13)
Vascular involvement	2 (3.13)
Clinical features throughout the course of BS	*N* (%)
Recurrent oral ulcers	64 (100.0)
Genital ulcers	49 (76.56)
Pseudofollicular lesions	26 (40.63)
Papulopustular lesions	19 (29.69)
Erythema nodosum	18 (28.13)
Ocular involvement	22 (34.38)
CNS involvement	4 (6.25)
Gastrointestinal involvement	28 (43.75)
Vascular involvement	13 (20.31)

*BS* Behçet’s syndrome*, CNS* central nervous system, *IQR* Interquartile range, *HLA* human leukocyte antigen, *SD* standard deviation

**Fig. 3:**
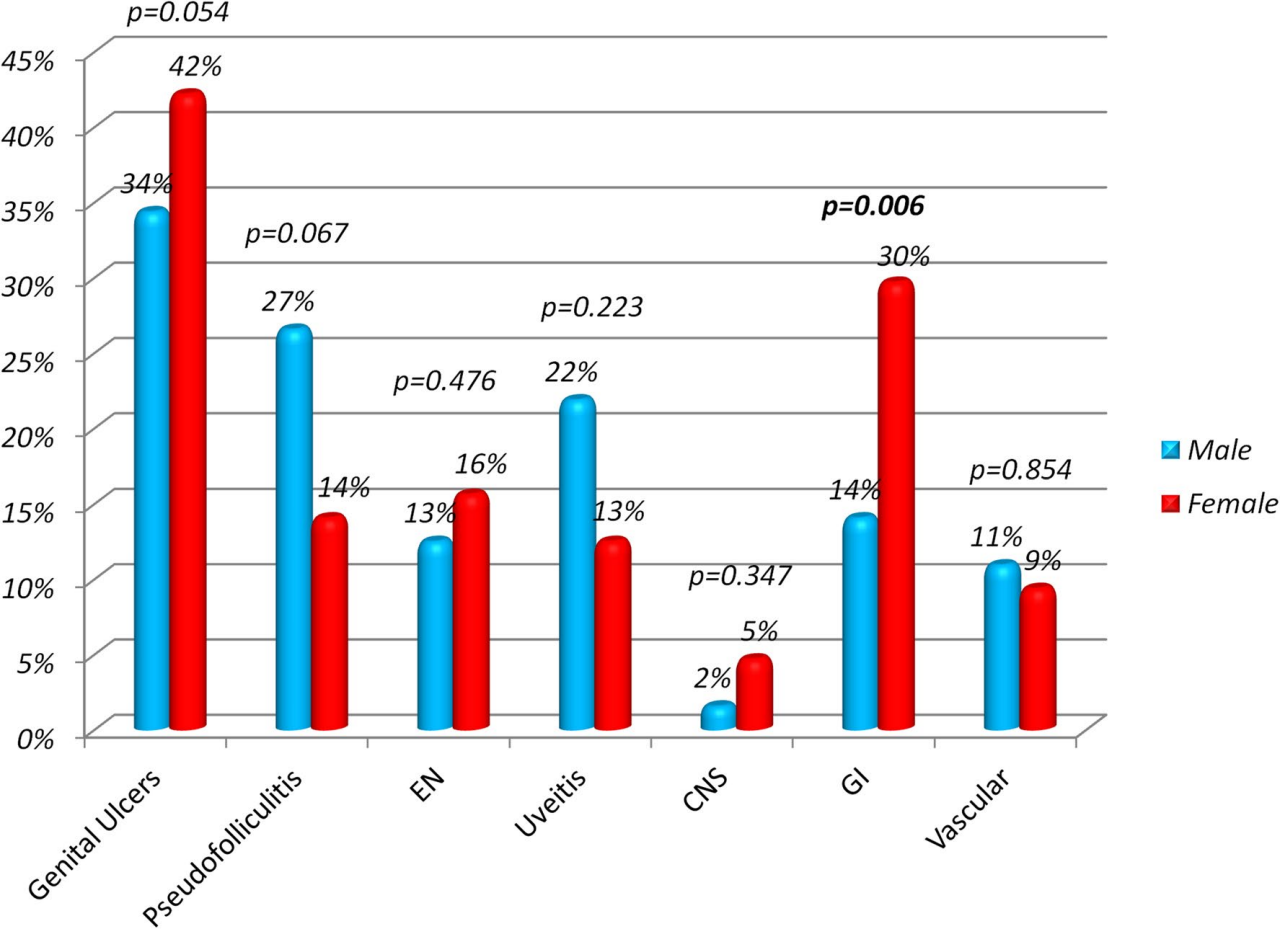
Comparison of gender differences (excluding oral aphthae) for 64 patients with juvenile-onset Behçet’s syndrome evaluated in our study. *EN* erythema nodosum, *CNS* central nervous system, *GI* gastrointestinal involvement

**Fig. 4:**
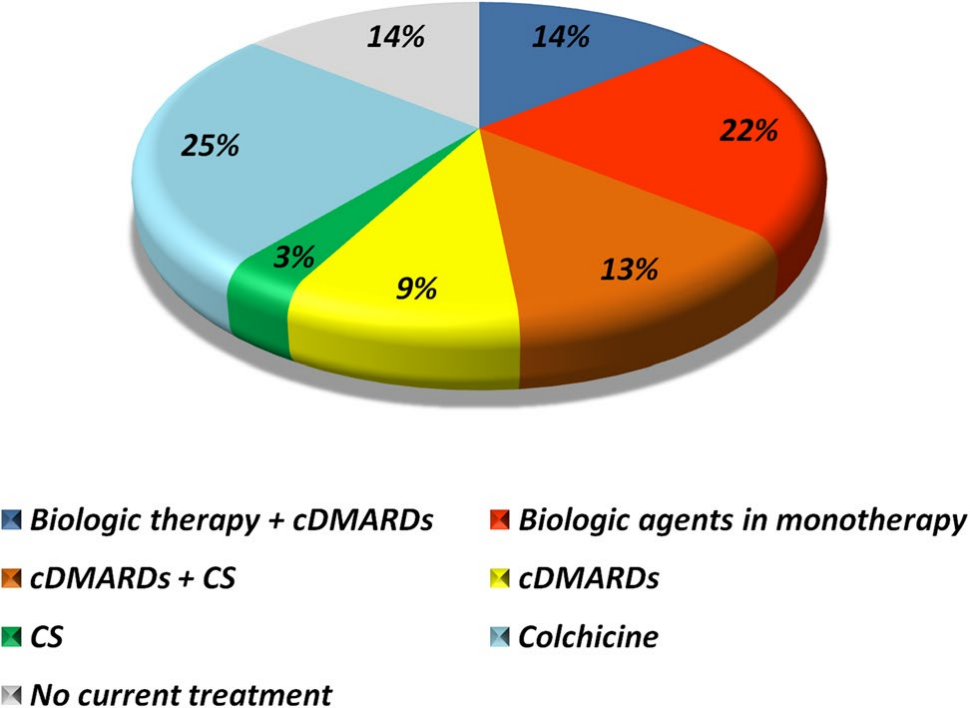
Therapeutic regimens administered in our cohort of 64 patients with juvenile-onset Behçet’s syndrome. *cDMARDs* conventional disease modifying anti-rheumatic drugs: *CS* corticosteroids

No gender differences emerged in the juvenile-onset cohort, except for nonspecific gastrointestinal signs encountered more frequently among female patients (*p* = 0.006). Gender differences are illustrated in Fig. 3. Concerning the therapeutic approaches, colchicine was the most frequently prescribed medication (25%), followed by biologics administered as monotherapy (22%) or in association with conventional disease modifying anti-rheumatic drugs (cDMARDs) (14%), and cDMARDs alone (13%). A considerable proportion of the juvenile-onset cohort (14%) did not receive any treatment at the last follow-up visit. A total of 23 BS patients with juvenile onset were treated with biologic agents. Nineteen of them received anti-tumor necrosis factor-α therapy, while the remaining four were administered anakinra (*n* = 2) and rituximab (*n* = 2). Figure 4 shows the treatment strategies used in the juvenile-onset population of our cohort.

### Comparisons of clinical manifestations between juvenile- and adult-onset BS and prognostic factors of disease severity

**Fig. 5:**
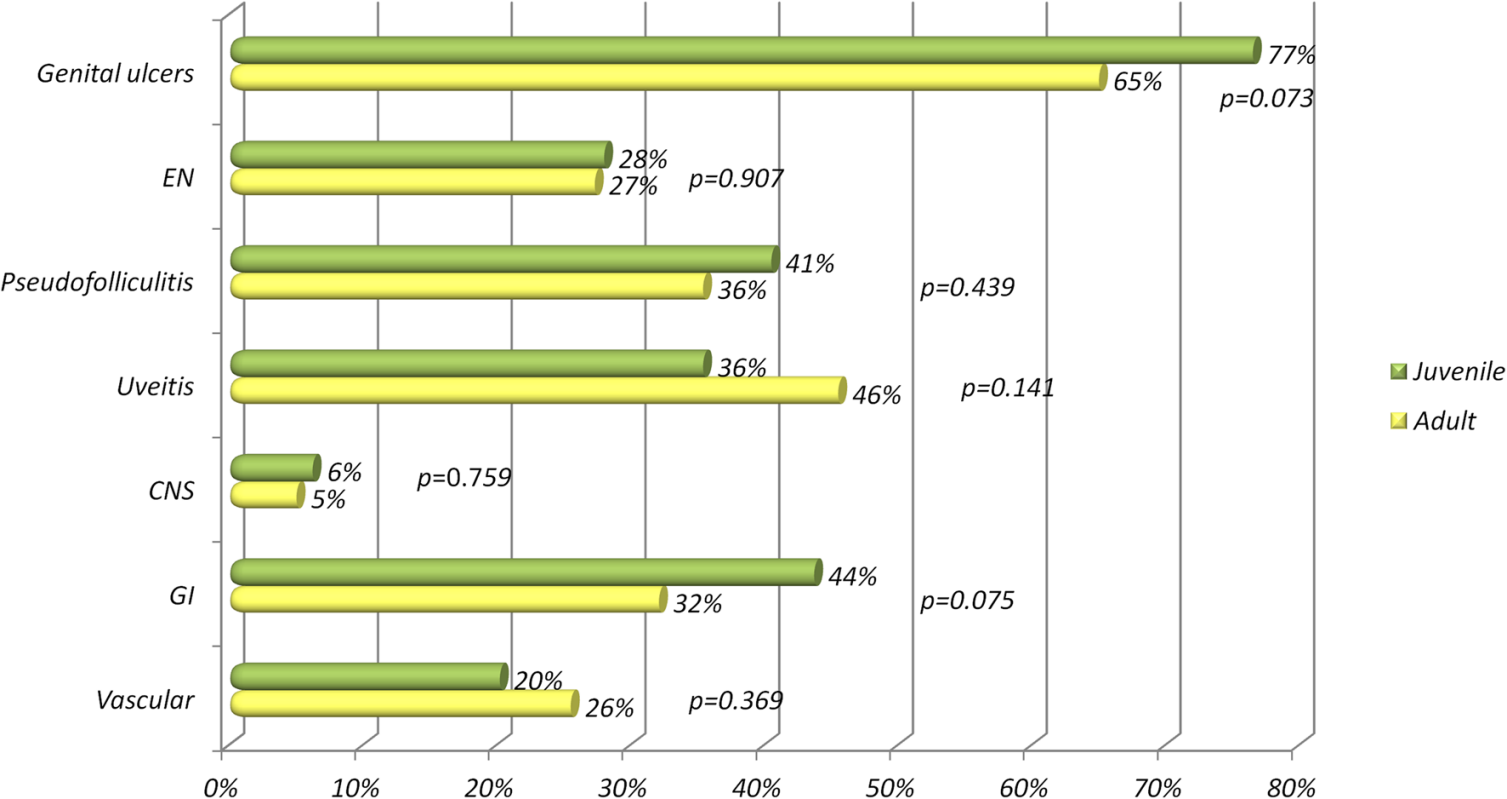
Clinical differences between juvenile-onset Behçet’s syndrome and adult-onset Behçet’s syndrome expressed in percentages for each manifestation (excluding oral aphthae) developed during the disease course. *EN* erythema nodosum, *CNS* central nervous system, *GI* gastrointestinal involvement

With regard to potential differences between juvenile-onset and adult-onset BS patients, HLA-B51 positivity was significantly more frequent among adult patients (*p* = 0.046). No statistical significant differences emerged neither for mucocutaneous signs, nor for specific major organ involvement. Figure 5 shows the percentages of every clinical sign developed during the disease course for juvenile- and adult-onset patients with BS. No differences were found between juvenile-onset and adult-onset BS with major organ involvement.

PEDBD criteria were applied in patients experiencing the first symptom before the age of 16, and 13 patients out of 64 fulfilled them. Throughout disease course, all patients had oral aphthosis (100%), nine of them genital ulcers (69%), eight patients presented pseudofolliculitis (61.5%), three papulopustular lesions (23.1%), three erythema nodosum (23.1%), seven (58.3%) uveitis, one (7,7%) central nervous system involvement, five (38.5%) gastrointestinal involvement and two (15.4%) vascular involvement. Multigroup comparisons between patients fulfilling the PEDBD criteria at onset, those with juvenile-onset and adult-onset, revealed a higher frequency of genital ulcers (*p* < 0.0001) and pseudofolliculitis and/or papulopustular lesions (*p* = 0.0065) in the pediatric group. With regard to other clinical manifestations, patients with juvenile-onset displayed a tendency toward a lower prevalence of uveitis (*p* = 0.018), without preserving statistical significance after post-hoc analysis (*Z* value 2.54).

**Fig. 6:**
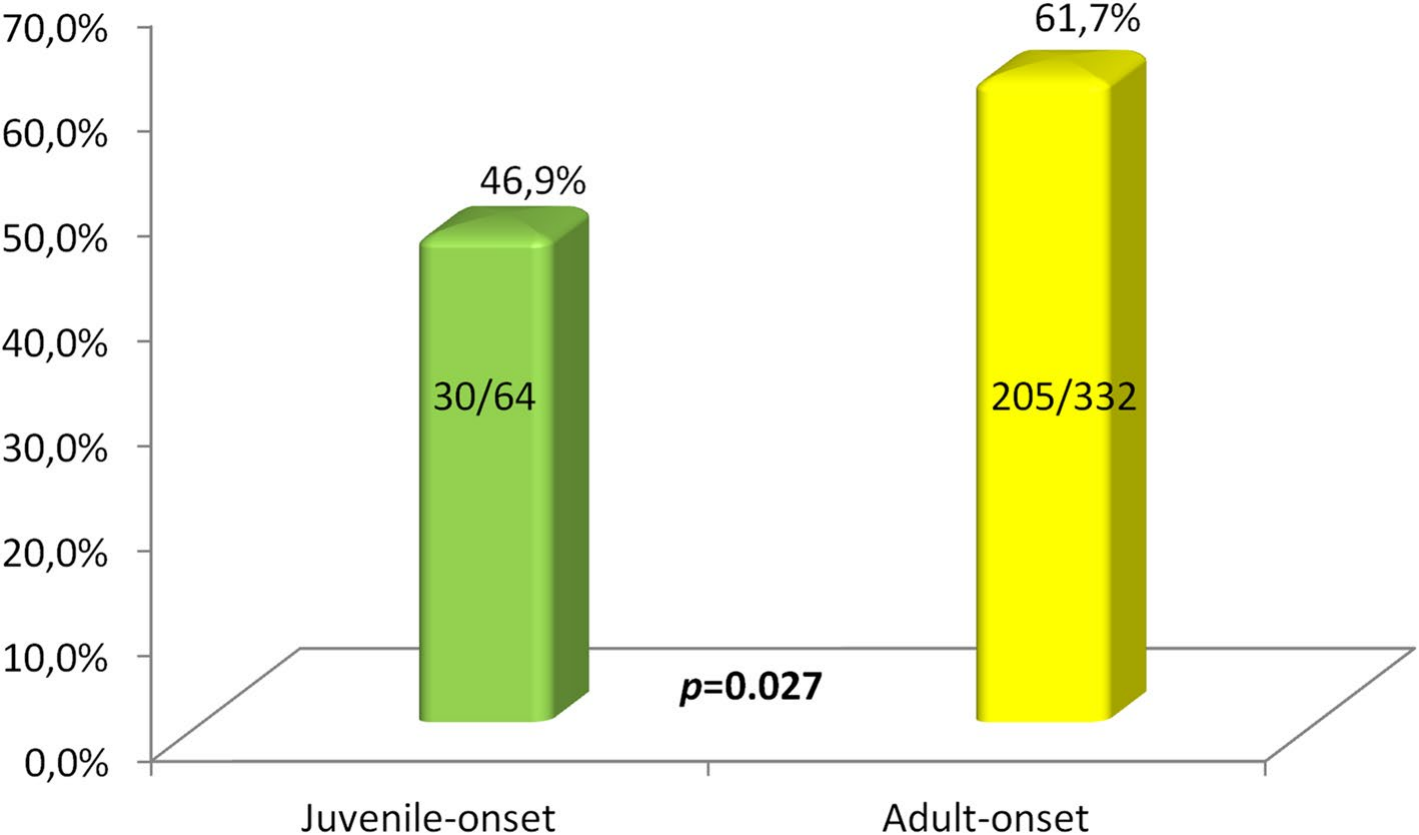
Differences in the development of major organ involvement between juvenile-onset and adult-onset Behçet’s syndrome throughout disease course

After the exclusion of nonspecific abdominal pain, juvenile-onset BS was less frequently associated with an overall major organ involvement (*p* = 0.027). The difference in the occurrence rate of major organ involvement between juvenile-onset and adult-onset BS is shown in Fig. 6. The last finding was corroborated also in the logistic regression conducted on the whole cohort, revealing that juvenile-onset leads to a reduced risk of developing long-term major organ involvement (OR 0.495 [0.263–0.932], *p* = 0.029). Other protective variables were the absence of major organ involvement at onset (OR 0.049 [0.019–0.126], *p* < 0.0001) and female gender (OR 0.477 [0.298–0.763], *p* = 0.002).

## Discussion

BS is an immune-mediated disorder with a relapsing–remitting nature and a marked clinical heterogeneity. This disorder affects patient’s quality of life, especially in some domains and if specific organs are involved [36]. Its clinical characteristics are well characterized in adults [13, 37], whereas pediatric data are still poor. Indeed, BS in children is rare and hard to recognize. Since the first reported case of pediatric BS [38], several efforts have been made to understand BS behavior in childhood [16, 17, 20–22]. However, comparisons with manifold studies are difficult and prone to biases given the unstandardized definition of juvenile-onset BS, since it has been used to indicate both age at onset of the first symptom and the fully manifested disease in the pediatric age [16, 17, 25, 26]. Diagnostic challenges represent another obstacle to overcome. Adult criteria like ISG criteria display a different performance than the PEDBD criteria, with a higher specificity but lower sensitivity [34, 39]. In the present study we report our experience on a relatively large cohort of juvenile-onset BS patients seen in three Italian university hospitals over a 2-year follow-up period.

Mean age at onset is concurrent with previous studies [16, 17, 21, 28, 40], despite a slightly lower age in other papers [22, 23, 25, 26, 30]. Also the male-to-female ratio is harmonic with the globally reported ratios [21–24, 27, 28]. HLA-B51 positivity was encountered in 50% of our juvenile-onset patients. Despite not being frequently tested in pediatric cohorts, this result is in agreement with the few studies reporting a frequency which ranges from 44 to 56.8% [18, 30, 39, 41]. A lower rate has been reported in German patients and a higher rate in Turkish patients [24] as well as in studies specifically investigating uveitis in juvenile cohorts, where HLA-B51 positivity has reached 75% [42, 43]. Interestingly, our group has recently reported long-term outcomes of BS-related uveitis, suggesting HLA-B51 as a predictor of long-term eye-related complications [44]. Familial cases were relatively high and similar to some previous studies [23, 30], but lower than in other reports [26]. Overall, the incidence of familial cases appears higher among patients with onset during childhood [24, 26, 30].

No differences in terms of clinical expression were detected between male and female patients. However, when nonspecific gut symptoms were also considered, males were outnumbered by female patients. It remains speculative whether this may be partially explained by a gynecological origin. Generally speaking, in contrast to what has been reported for adult cohorts [11, 13], gender does not seem to have a major impact in the clinical expression of juvenile-onset BS.

With regard to clinical differences between juvenile- and adult-onset patients, results were comparable for mucocutaneous signs and specific major organ involvement both at disease onset and at the last follow-up visit. The spectrum of juvenile-onset BS resembled that of the adult counterpart. In support of this finding, a recent Egyptian study of 1562 adults with BS, also including 91 patients (17.2%) with juvenile-onset, did not show any differences in disease manifestations between young and adult patients [19]. This may suggest that differences in pediatric and adult cohorts may be related to disease duration rather than age of onset. It is also possible that the advent of more targeted and effective therapies may halt the natural disease course and narrow down the gap under a clinical viewpoint between these two subgroups. Indeed, one third of our cohort were treated with biologic agents. In this regard, targeted therapies with biologics have proven to be valid options on both specific organ involvement such as intraocular inflammation [44–47] as well as in controlling all protean manifestations of BS and its activity [48, 49].

When pediatric, juvenile- and adult-onset BS patients were compared, we found a higher frequency of genital ulcers, pseudofolliculitis and/or papulopustular lesions in the pediatric group and a lower prevalence of uveitis in the juvenile-onset population. Contrarily to what has been previously reported by other authors [16, 17, 25, 50], we detected a higher frequency of genital aphthae among BS patients with juvenile-onset. One of the most ominous manifestations in BS is intraocular inflammation: it affects a considerable proportion of patients ranging from 30 to 70% and is a prominent cause of morbidity [11, 12, 51]. Controversial results have been published in this topic with studies displaying a higher frequency in children [52] and others reporting a lower frequency [22, 23]. In our sample uveitis followed a trend toward a lower prevalence among juvenile-onset patients when compared to adult-onset ones and to those in whom the disease was fully manifested before the age of 16. In addition, BS does not appear to be a common cause of pediatric uveitis, even in endemic regions where BS prevalence is quite high [50].

Juvenile-onset patients in several countries were reported to have significantly more frequent gastrointestinal complications and nonspecific gastrointestinal symptoms than adult-onset ones [16, 22, 23, 25, 28]. According to our data, complications were rare in juvenile-onset patients and no differences emerged between them and adult patients. Therefore, based on our findings, an age-driven influence in BS phenotype seems unlikely. Clinical differences among various studies reflect a pronounced geographic heterogeneity of BS. Discrepancies among countries, including the present one, may be a consequence of strong ethnic variability. Additionally, they may also be a product of different methodological approaches based on sampling, type of examinations or even the distinction of juvenile and pediatric BS as well as the set of criteria employed to formulate such diagnosis.

Although the clinical spectrum looks quite similar, the frequency of major organ involvement and disease severity along with prognosis may differ according to the age at onset. Unlike patients affected by systemic lupus erythematosus, for whom there is a robust evidence showing a more severe course of juvenile-onset disease compared to adults [53], data on BS are scarce and controversial [15, 24, 25, 29] with insufficient reports addressing the impact of age for prognosis in children. In our series, juvenile-onset patients were under-represented in terms of major organ involvement when nonspecific abdominal pain was excluded, suggesting a better prognosis in those patients experiencing the first disease manifestation before the age of 16. In support of the latter, different authors have described a less severe course [20, 25] and a lower rate of severe complications, notably blindness, among juvenile-onset patients [24]. Furthermore, the less common HLA-B51 positivity in our juvenile-onset cohort, considered by some authors as a marker of poor prognosis, provides additional support to explain a less severe course. It is also tempting to speculate that the lower HLA positivity along with a higher familial aggregation [23, 24, 26, 30] may reflect a distinct genetic background and ultimately an overall different pathogenetic pathway in pediatric as well as in juvenile-onset patients.

However, it is still unclear which factor may favor early expression and impact disease severity during BS course. A higher degree of awareness is needed also among general practitioners. A multidisciplinary approach in collaboration with pediatricians, ophthalmologists, neurologists and other specialists is mandatory in order to uncover some of the unanswered questions. Our findings should be contextualized on the basis of some limitations such as the retrospective design of the study. To this end, describing predictors or prognostic factors would be ambitious and our results should be interpreted cautiously. The relative homogeneity of the cohort of patients may limit the possibility of generalizing any conclusions. Moreover, given the lack of standardized diagnostic and/or classification criteria for the pediatric age at the time of disease onset in most of our patients, as well as the lack of validated outcome measures in children complicates any comparisons with other studies.

In conclusion, the present study details the clinical picture of juvenile-onset BS and represents an additional attempt to shed light in this understudied topic. In our cohort, juvenile-onset BS resembled more or less the same clinical spectrum of adult-onset patients, without any influence of gender. The clinical expression does not vary significantly according to age, and gender does not influence the clinical phenotype among juvenile-onset patients. Therefore, these variables should not be driving factors in the treatment approach or in the follow-up management. Patients with a full-blown disease at onset in the pediatric age display more frequent mucocutaneous manifestations. Furthermore, young patients seem to have a less severe course by developing less frequently major organ involvement. As a matter of fact, patients experiencing their first symptom before 16 years have a tendency to develop less frequently uveitis and to a broader extent major organ involvement, which should suggest a better prognosis on the long run for this subgroup. These findings may have important therapeutic implications, but more robust and well-designed studies are needed to draw firm conclusions.

## Data Availability

The datasets generated for this study are available on request to the corresponding author.
